# Preoperative imaging strategies to optimize surgical treatment in primary hyperparathyroidism (pHPT)

**DOI:** 10.1186/s12893-026-03768-5

**Published:** 2026-05-26

**Authors:** Felicitas Fries, Jennifer Thaeren, Susanne Greschus, Eugen Muschler, Holger Palmedo, Haug-Lambert Loriz, Nicola Cerasani, Andreas Türler, Stefan Klozoris, Udo Schmitz, Glen Kristiansen, Kai Wilhelm

**Affiliations:** 1 Department of Radiology and Nuclear Medicine, Johanniter Hospital Bonn, Johanniterstraße 3-5, Bonn, 53113 Germany; 2 Institute of Radiology and Nuclear Medicine, Kaiser-Passage Bonn, Bonn, 53113 Germany; 3 Department of General and Visceral Surgery, Johanniter Hospital Bonn, Bonn, 53113 Germany; 4 Department of Internal Medicine, Endocrinology, Johanniter Hospital Bonn, Bonn, 53113 Germany; 5 Institute of Pathology, Bonn University Hospital, Bonn, 53127 Germany

**Keywords:** Primary hyperparathyroidism, Parathyroid adenoma, Parathyroidectomy, Diagnostic imaging, Ultrasound, Tc-99m-MIBI, SPECT/CT, 99m Tc-sestamibi SPECT/CT, MRI, 18-F PET/CT, Selective venous sampling (SVS)

## Abstract

**Purpose:**

Accurate preoperative localization of parathyroid adenomas is essential to enable targeted and minimally invasive surgery in patients with primary hyperparathyroidism (pHPT). A variety of imaging techniques are available for this purpose. In routine clinical practice, cervical ultrasound (US) combined with scintigraphy is most commonly used as the initial diagnostic approach. However, the optimal strategy or combination of imaging modalities for reliable localization prior to surgery and its relevance for postoperative outcomes remains a matter of ongoing discussion. The aim of this study was therefore to evaluate the diagnostic performance of different preoperative imaging modalities and their combinations for the detection and localization of parathyroid pathology in a large cohort of patients treated at a specialized thyroid center. This study investigated how well the values reported under controlled study conditions translate to clinical practice and real-world application.

**Methods:**

This retrospective study included 325 patients who underwent minimally invasive parathyroidectomy for primary hyperparathyroidism between January 2015 and December 2023. All patients were evaluated with respect to the preoperative imaging modalities used for localization of the pathological parathyroid gland. In most cases, the standard diagnostic approach consisting of cervical US and scintigraphy was performed initially. When these examinations did not provide conclusive localization, additional imaging techniques were applied, including magnetic resonance imaging (MRI), computed tomography (CT), or 18-fluorocholine PET-CT (18-F PET-CT). In selected cases, invasive selective venous blood sampling (SVS) was also performed.

The diagnostic performance of the different imaging modalities was assessed with regard to correct preoperative localization of the adenoma. Furthermore, the influence of factors such as concomitant thyroid disease, previous thyroid or parathyroid surgery, adenoma size and weight, as well as biochemical parameters including calcium and parathyroid hormone levels before, during, and after surgery was analyzed.

**Results:**

The primary endpoint of postoperative normalization of parathyroid hormone levels after minimally invasive parathyroidectomy was achieved in 94.2% of patients undergoing surgery. The combination of US and scintigraphy as the primary examination procedures was able to provide clear information regarding the localization of the adenoma in 51% of cases, in all other cases one or more additional imaging procedure had to be performed. On average, three examination modalities (triple localization methods) had to be performed per patient (IQR 2.00 - 3.00), with MRI as the most common supplemented procedure, so that in the end a median of two examinations (IQR 1.00 - 3.00) consistently indicated the correct localization. Related to the individual method, US had the highest over-all sensitivity (69.4%) of the imaging procedures. Scintigraphy and SPECT-CT had a similarly high sensitivity of 58.0% for scintigraphy and 56.4% for SPECT-CT. MRI still achieved a sensitivity of 52.9%, CT 36.6%, 18-fluocholine PET-CT 100% and selective venous blood sampling only 60%. The values in clinical practice and broader real-world application with exception of 18-fluocholine PET-CT were below those reported under controlled study conditions.

US, scintigraphy, SPECT-CT and MRI each showed a decrease in the sensitivity of correct preoperative localization in the presence of simultaneous thyroid disease. Selective venous blood sampling showed a drop in sensitivity from 80% without prior surgery to 28.6% with prior thyroid or parathyroid surgery.

The mean values of correctly selected parathyroid adenomas suggested that larger and more heavy parathyroid adenomas were more likely to be recognized. It was shown that parathyroid adenomas examined by US, scintigraphy, SPECT-CT and additional MRI had a significantly lower volume (*p* = 0.004) and weight (*p* = 0.045) than those examined by US and scintigraphy alone.

The possibility of successful preoperative localization did not depend on the specific parathyroid hormone level. In contrast, it was shown that higher preoperative calcium levels do not necessarily correlate with easier imaging detection, but lower calcium levels are more often associated with greater diagnostic effort. In contrast, preoperatively determined parathyroid hormone correlated moderately with adenoma weight (*r* = 0.44; *p* < 0.001) and adenoma volume (*r* = 0.17, *p* = 0.024), whereas calcium showed only weak, albeit significant, correlations with adenoma weight (*r* = 0.20; *p* = 0.005) and volume (*r* = 0.17; *p* = 0.024).

Without concomitant thyroid disease, the US determined volume correlated very strongly with the histopathologically determined volume with a Spearman correlation coefficient of 0.702 (*p* <.001). With concomitant thyroid disease, the Spearman correlation coefficient decreased to 0.60 (*p* <.001), although there was still a strong correlation.

**Conclusion:**

The findings of this study indicate that the combination of cervical ultrasound and scintigraphy remains an effective first-line imaging strategy for the localization of parathyroid adenomas in the majority of patients undergoing surgery for primary hyperparathyroidism in routine clinical practice. This approach was associated with a high success rate of minimally invasive parathyroidectomy in a specialized thyroid center. Nevertheless, a considerable proportion of patients required additional imaging modalities to achieve reliable preoperative localization, with MRI representing the most frequently used supplementary technique.

## Introduction

Primary hyperparathyroidism (pHPT) is a common endocrine disorder and is characterized by inappropriate secretion of parathyroid hormone accompanied by elevated serum calcium levels. The reported incidence ranges between 20 and 30 cases per 100,000 individuals annually. In the majority of patients, the disease is caused by a single parathyroid adenoma (80%), whereas multiple or ectopic adenomas (5%), parathyroid hyperplasia (15%), or rarely parathyroid carcinoma represent less common etiologies [1, 2]. In addition, hereditary forms of the disease may occur in association with genetic syndromes such as multiple endocrine neoplasia types 1 and 2 A [3, 4].

Clinical manifestations of pHPT are heterogeneous. Classical complications include nephrolithiasis, peptic ulcer disease, and skeletal involvement such as osteoporosis [5]. There are other less frequent symptoms including cardiovascular problems (hypertension, calcifications of the heart valves and myocardium, hypertrophy of the left ventricle and shortening of the QT segment, which favors arrhythmias and increased mortality from acute myocardial infarction and stroke) and neuropsychiatric conditions (confusion, depression, cognitive impairment, sleep disorders, irritability or decreased concentration). In many industrialized countries, however, pHPT is increasingly detected in patients without obvious symptoms as a result of routine biochemical screening [6].

Surgical removal of the hyperfunctioning parathyroid gland remains the only curative treatment option. Parathyroidectomy is therefore considered the standard therapy for patients who meet surgical criteria in order to prevent disease progression and long-term complications. Both symptomatic and asymptomatic patients may benefit from surgical intervention, with several studies reporting improvements in quality of life after successful treatment [7–9].

Advances in surgical techniques have led to the development of minimally invasive approaches, including open minimally invasive parathyroidectomy (OMIP), minimally invasive radio-guided parathyroidectomy (MI-RP), minimally invasive video-assisted parathyroidectomy (MIVAP), and fully endoscopic techniques. Among these procedures, MIVAP represents one of the most commonly used methods.

Various imaging techniques have been developed to identify parathyroid adenomas prior to surgery. In clinical practice, multiple modalities are often used in combination because no single method provides perfect diagnostic accuracy. Accurate localization is particularly important to facilitate focused surgical approaches while minimizing operative trauma for the patient [10].

Nevertheless, incorrect or inconclusive imaging findings may occur, and anatomical variability of the parathyroid glands can lead to unsuccessful initial surgery. In such cases, further surgical exploration, including bilateral neck exploration (BNE) or even mediastinal approaches, may become necessary [11].

Current clinical practice commonly involves the use of at least two imaging modalities during the preoperative work-up, with reported surgical success rates of up to 95%. Despite these favorable outcomes, there is still no clear agreement regarding the optimal diagnostic strategy or the most effective combination of imaging techniques [3]. The recommendations of the American Association of Endocrine Surgeons therefore emphasize that the selection of imaging studies should be guided by experienced clinicians who are familiar with local diagnostic capabilities (strong recommendation; low-quality evidence) [12].

The present study aimed to evaluate the diagnostic performance of different preoperative imaging modalities and their combinations for the localization of parathyroid pathology in a large cohort of patients undergoing surgery at a specialized thyroid center. Particular emphasis was placed on the accuracy of localization as well as the relationship between imaging findings and postoperative outcomes.

## Materials and methods

This retrospective analysis involved 325 patients who received MIVAP on at least one parathyroid gland for pHPT from January 2015 to December 2023, focusing on various preoperative imaging modalities. This retrospective observational study was conducted in accordance with the ethical principles of the Declaration of Helsinki and the applicable institutional regulations [13]. Ethical approval was not required according to the regulations in place at the time the study was conducted. This retrospective study does not necessitate clinical trial registration per ICMJE standards.

The patients were identified via a systematically maintained database. Clinical data, imaging findings, laboratory values, and histopathological reports were extracted from the institutional electronic medical record system. The surgical site was determined by examining the procedure record. The outcomes of preoperative imaging techniques were compared with surgical results. Patients with absent or ambiguous histological data, lacking surgical site information, or missing radiological data were eliminated.

In routine clinical practice, cervical US combined with scintigraphy served as the initial imaging strategy for all patients. In cases where localization was ambiguous, routine diagnostics were augmented with additional techniques, including MRI, CT, or 18-F PET-CT, and in other instances, invasive SVS was employed. The various imaging modalities were evaluated for their efficacy in accurately preoperatively localizing the adenoma, alongside considerations of concurrent thyroid pathology, prior thyroid and parathyroid surgeries, the dimensions and mass of the excised parathyroid adenoma, and biochemical parameters including calcium and parathyroid hormone levels both pre- and postoperatively.

Seventeen individuals who underwent parathyroidectomy were excluded due to the absence of histological evidence (82.4%) or as a result of an intraoperative incidental finding (17.6%). The investigative cohort comprised 308 individuals, which were categorized into two groups: 294 patients who received surgery on a single parathyroid gland and 14 patients who underwent simultaneous parathyroid surgery on several glands. This study focused on patients who received surgery on a single parathyroid gland.

Following preoperative diagnostics, the patients underwent minimally invasive surgery using MIVAP as pioneered by Miccoli et al. [14]. If indicated preoperatively, a supplementary hemithyroidectomy or thyroidectomy was also performed either using a minimally invasive technique such as MIVAT as described by Bellantone et al. [15] and Miccoli et al. [16] or conventional thyroidectomy.

In instances where histological investigation identified abnormal parathyroid tissue, the surgical site was deemed the gold standard for comparison with the imaging location. Surgery was regarded as successful when an abnormal parathyroid gland was detected during the procedure, excised, and later confirmed histopathological. The term pathological parathyroid tissue referred to parathyroid adenoma, hyperplasia, oxyphil, or carcinoma [17].

Concordance between imaging findings was assessed by comparing the anatomical localization obtained from imaging with the intraoperative location documented in the surgical report. Anatomical locations were grouped into the following categories: left central, left upper, left lower, right central, right upper, right lower, mediastinal, ectopic or non-localizable.

All US examinations were conducted internally by a team of specialized head and neck radiologists, possessing considerable expertise in parathyroid imaging. The combination of US and scintigraphy offers precise preoperative localization for individuals with primary hyperparathyroidism. Owing to the current billing framework and allocation attributes of the thyroid center, all supplementary imaging procedures were conducted on an outpatient basis across various referral practices.

**Fig. 1:**
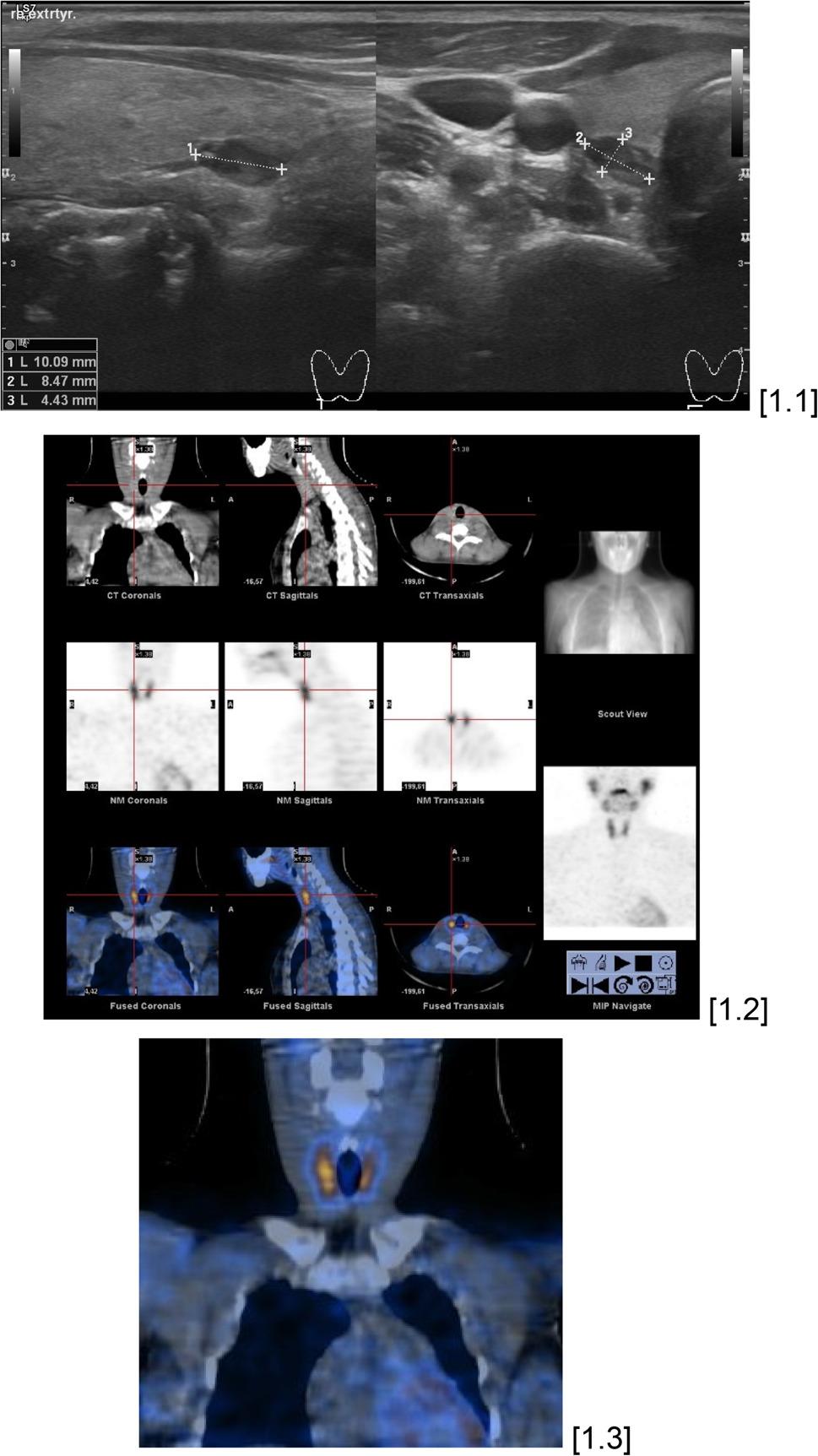
33-year-old female patient suffering from asymptomatic primary hyperparathyroidism without concomitant thyroid disease. Both the US [1.1] and the SPECT-CT [1.2, 1.3] were able to consistently demonstrate the localization of the adenoma on the right caudal side with a volume of approximately 0,2 ml. The preoperatively measured parathyroid hormone level was 130.7 pg/ml and the preoperatively measured calcium level was 2.85 mmol/l. Intraoperatively, there was an adequate drop in the parathyroid hormone level to 52.7 pg/ml, which correlates with an intraoperative drop of about 60%. Postoperatively, the parathyroid hormone level remained adequate at 35.8 pg/ml and the calcium level was 2.4 mmol/l. Histopathology confirmed hyperplastic parathyroid tissue compatible with a parathyroid adenoma

All patients received in-house high-resolution US at 7.5 MHz for the thyroid and parathyroid glands, along with the soft tissue of the neck, utilizing a linear transducer. Scintigraphy was conducted either by the planar approach or, in most instances, utilizing SPECT scintigraphy. In 36.7% (*N* = 108) of the patients, scintigraphy was augmented by SPECT-CT (Fig. 1). The image creation in the SPECT-CT was conducted using either a hybrid approach or offline software fusion.

For individuals in whom these two imaging modalities failed to yield a definitive localization, further exams were conducted. A supplemental MRI is typically conducted using a 1.5T magnet with a head-neck coil, incorporating at least one axial, sagittal, and coronal plane featuring T1 and T2 mDixon sequences, alongside T1 and T2 TSE sequences following intravenous contrast injection, as well as DWI and perfusion imaging.

For individuals in whom these supplementary imaging techniques failed to yield a definitive localization or in cases where MRI was contraindicated, an alternative imaging modality was employed. The patients received contrast-enhanced CT with multiplanar reconstruction, including at least one native, arterial, and venous phase.

**Fig. 2:**
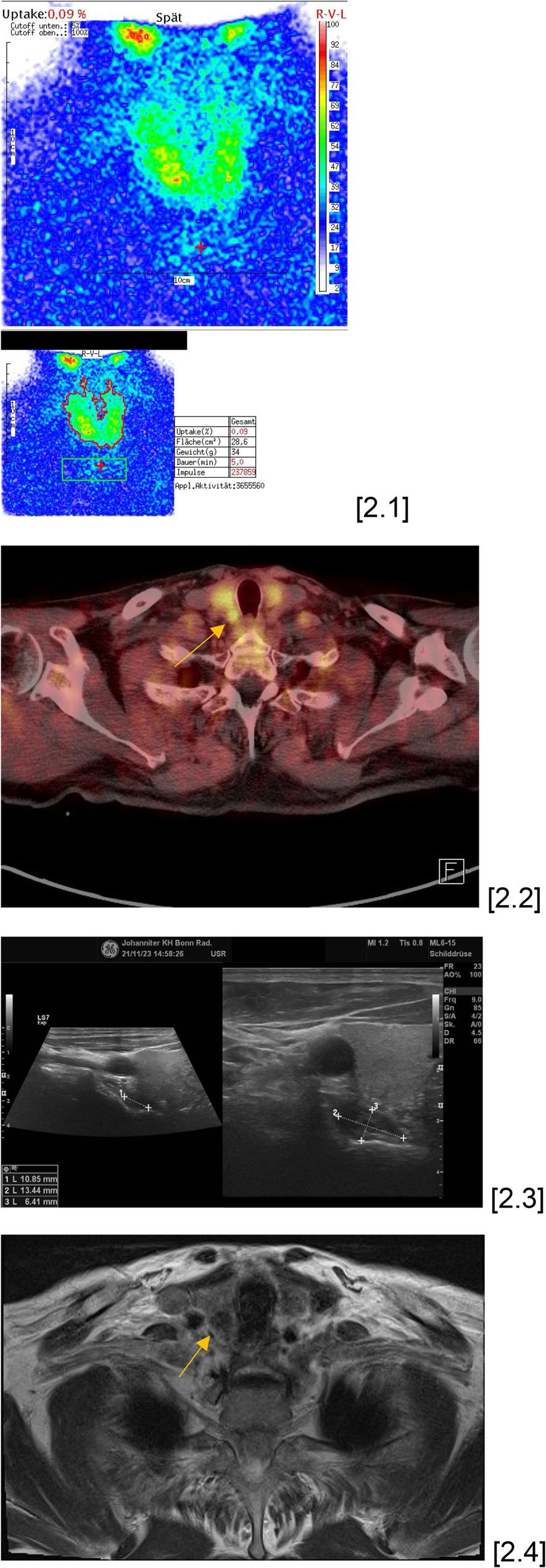
76-year-old male patient suffering from primary hyperparathyroidism with manifest osteoporosis. No parathyroid adenoma could be detected by scintigraphy [2.1]. The supplementary performed PET-CT revealed an adenoma localization on the right caudal side [2.2]. This adenoma localization was confirmed both in the additional US examination and in the MRI scan [2.3, 2.4]. Intraoperatively, the parathyroid adenoma was found caudally on the right, as suspected, and after successful parathyroidectomy there was an intraoperative drop in parathyroid hormone from 131.8 pg/ml to 28.9 pg/ml, which correlates with an intraoperative drop of about 78%. Postoperatively, the parathyroid hormone level remained adequate at 35.4 pg/ml and the calcium level was 2,45 mmol/l. Histopathological examination revealed nodular parathyroid tissue, consistent with a parathyroid adenoma

The least frequently performed non-invasive procedure, considering availability and examination costs, was to conduct an 18-F PET-CT. Following the administration of F-18 choline, positron emission tomography (PET) was conducted one hour post-injection, producing data in transverse, sagittal, and coronal slices. Furthermore, a spiral CT scan of the full body trunk was conducted using native technology, resulting in the development of PET-CT fusion pictures in all three planes (Fig. 2).

An invasive SVS was conducted as a last measure for the lateral location of the hormone-secreting parathyroid gland or ectopic adenoma. Buckley and colleagues provide a comprehensive description of the process and specialized approach in SVS for parathyroid tumors. The SVS examinations were all carried out at our thyroid centre as part of the treatment and thus followed the standardized procedure prescribed by Buckley and colleagues [18, 19].

For further investigation the study population was divided into a scintigraphy and a SPECT-CT group according to the preoperative localization diagnostics obtained. In addition, the combinations of different imaging procedures used were analyzed with regard to their frequency. Combinations constituting ≥ 5% of the total examinations conducted were analyzed concerning the different endpoints. Preoperative localization information was deemed positive for all operations if both horizontal and vertical localization were accurately anticipated as per the surgical report.

The principal outcomes included the surgical reduction in parathyroid hormone levels and the normalization of calcium levels in the discharge laboratory results. The additional endpoints of our study included an accurate prediction of adenoma volume and weight. Where feasible, the volumes assessed preoperatively via US, MRI, and CT were compared with the volumes derived from histological results. The volumes were computed utilizing the ellipsoid formula (volume of the parathyroid adenoma = (length x breadth x height) x 0.52).

### Statistics

The processing and evaluation of the underlying study data was carried out using IBM SPSS Statistics 29 (IBM Corporation, USA) on Mac. Firstly, the data was analyzed using descriptive statistics. Kolmogorov-Smirnov and Shaphiro-Wilk tests were performed to test normal distribution of the data. Group comparisons were performed using Student’s t-test, Chi squared test as well as contingency tables with the determination of the conformity measure Cohen’s kappa. Possible associations with clinical parameters and the examination using SPECT-CT or scintigraphy were analyzed using the Kruskal-Wallis test and the Mann-Whitney-U-Test. The Spearman rank correlation coefficient was used for possible correlations. The correlation was considered strong in a range from 0.5 to 0.7 and very strong from 0.7 to 1.0. Differences were assumed to be statistically significant at *p*-values ≤ 0.05.

## Results

From the group of 294 patients, who underwent surgery of one parathyroid gland, 236 were women (80.3%) compared to 58 men (19.7%). The mean age at onset of the disease was 58 years with a wide range from a minimum of 19 years to a maximum of 86 years. Symptomatic PHPT disease including kidney stones, osteoporosis or ulcers only occurred in 30.6% of the patients.

More than half of the operated patients suffered from concomitant thyroid disease (51%) and 124 patients underwent simultaneous thyroid and parathyroid surgery (42.2%). In 11.9% of patients (*N* = 35), previous neck surgery had already been performed at the time of surgery. Of these, 2.7% (*N* = 8) had previously undergone parathyroid surgery, which was unsuccessful in 1.0% (*N* = 3) of cases. In 1.0% of cases (*N* = 3), a mediastinal parathyroid adenoma was the cause of primary hyperparathyroidism. In addition, there were 0.3% ectopic parathyroid adenomas (*N* = 1) in this case, left paraesophageal and 1.7% (*N* = 5) parathyroid adenomas located in the thyroid gland. Hyperparathyroidism was only caused by parathyroid carcinoma in 0.3% (*N* = 1) of the patients.

Most patients (96.3%), were able to undergo surgery using MIVAP (*N* = 283). In the case of a parathyroid adenoma left paraesophageal the surgical management did not differ from MIVAP. In the case of parathyroid carcinoma, intraoperative suspicion of malignancy was raised based on the macroscopic appearance and anatomical situation. Frozen section analysis supported the suspicion of carcinoma. Consequently, an immediate completion surgery consisting of ipsilateral hemithyroidectomy and neck dissection was performed.

In cases with suspected intrathyroidal parathyroid adenomas based on preoperative imaging, a minimally invasive video-assisted hemithyroidectomy (MIVAT) was performed. Intraoperative frozen section analysis was used to confirm parathyroid tissue, and intraoperative parathyroid hormone monitoring was applied to document an adequate biochemical response.

The mediastinal adenomas identified in this study were located in the upper mediastinum and were accessible via a cervical approach; therefore resection was performed without the need for thoracic procedure, such as video-assisted thoracoscopic surgical (VATS) or sternotomy.

The median preoperative parathyroid hormone (PTH) level was 128.80 pg/ml (range 2.5–2077.0 pg/ml; reference range 15–65 pg/ml) and the median postoperative PTH level was 27.3 pg/ml (range 1.4–95.3 pg/ml). The primary endpoint of the study, i.e. post-operative normalization of parathyroid hormone levels, was achieved in 94.2% (*N* = 277) of all patients who underwent surgery. The median preoperative calcium level was 2.77 mmol/l (range 2.36–3.48 mmol/l; reference range 2.15–2.5 mmol/l) and the median postoperative calcium level was 2.33 mmol/l (range 1.79 to 2.75 mmol/l). Normalization of calcium levels postoperatively was achieved in 78.6% of patients (*N* = 231). In 8.5% of cases (*N* = 25), hypocalcaemia occurred postoperatively. Only 12.9% of patients (*N* = 38) continued to have hypercalcaemia.

A total of 860 imaging examinations were performed on patients undergoing surgery for PHPT prior to the operation.

All patients underwent in-house US of the neck. In addition, 97.3% of the patients received a scintigraphy (*N* = 286), which was carried out by different practices or clinics. Scintigraphy was performed in 17.8% (*N* = 52) using the planar technique and the majority of 82.2% (*N* = 240) as scintigraphy with SPECT. Only a minority of 1.0% of the scintigraphies (*N* = 3) used a subtraction technique. In 9.9% of patients (*N* = 29), parathyroid adenomas were found during thyroid scintigraphy. In 37.4% (*N* = 110) of the patients, the scintigraphy was supplemented by a SPECT-CT. The SPECT-CT findings were available as fusion findings in 66.3% (*N* = 73), only in 30% (*N* = 33) as single findings and in 1.8% (*N* = 2) of the cases no written findings were available.

A supplementary MRI scan was performed in 39.1% of patients (*N* = 115) and a CT scan in 14.0% (*N* = 41). An invasive SVS as preoperative localization diagnostics was performed in only 4.1% of cases (*N* = 12). The 18-F PET-CT, which is not yet clinically established, was used in only 1.36% of patients (*N* = 4).

Using US, 69.4% of the localizations of the parathyroid adenomas could be correctly predicted. Scintigraphically, this proportion was still 58.0% in our study and thus slightly above the correctly predicted proportion of 56.4% in SPECT-CT. This was followed closely by MRI, in which this proportion was still 52.9%. SVS as an invasive examination was able to predict the correct localization in 50.0% of cases. Using CT imaging, the correct position could only be predicted in 36.6% of cases (Table 1).

**Table 1:** Comparison of the frequencies of the different examination modalities performed and their sensitivity

Imaging performed	Total number of examinations (*N*)	Correct localization (*N*)	Sensitivity (%)
US	294	204	69.4
scintigraphy	286	166	58.0
SPECT-CT	110	62	56.4
CT	41	15	36.6
MRI	119	63	52.9
SVS	12	6	50.0
18-F PET-CT	4	4	100.0
total	866	520	60.0

In the following, the localization of the parathyroid adenomas correctly predicted preoperatively using the different localization diagnostics was analyzed separately for a group without and with concomitant thyroid surgery (Table 2). It was shown that there was a significant reduction in sensitivity for all standard procedures established in clinical practice. The only exceptions were the 18-F PET-CT and the SVS, albeit with only a small number of cases.

**Table 2:** Comparison of imaging procedures performed in patients without and with concomitant surgery for thyroid disease by number and percentage of correct examinations out of the total number of examinations performed with this procedure. Only the SVS and the 18-F PET-CT showed no decrease in sensitivity in the presence of concomitant thyroid pathology with indication for surgery. All other imaging procedures showed a decrease in sensitivity in these patients

Imaging performed	without concomitant thyroid surgery			with concomitant thyroid surgery		
	Total number of examinations (*N*)	Correct localization (*N*)	Sensitivity (%)	Total number of examinations (*N*)	Correct localization (*N*)	Sensitivity (%)
US	170	134	78.82	124	70	56.45
scintigraphy	163	104	63.80	123	62	50.41
SPECT-CT	63	40	63.49	47	22	46.81
CT	17	8	47.06	24	7	29.17
MRT	60	37	61.67	59	26	44.07
SVS	4	2	50.00	8	4	50.00
18-F PET-CT	3	3	100	1	1	100
total	480	328	68.33	385	191	49.61

 It could be shown that US, MRI and angiography are more affected by previous surgery in terms of sensitivity than CT, scintigraphy, SPECT-CT and 18-F PET-CT (Table 3).

**Table 3:** The dependence of the sensitivity of the different preoperative imaging modalities on previous thyroid or parathyroid surgery. US, MRI and SVS showed a decrease in sensitivity. Scintigraphy and SPECT-CT showed no major influence with regard to their sensitivity depending on previous operations on the thyroid or parathyroid gland

procedure	No previous surgery			Previous surgery		
	*N*	Correctly predicted	Sensitivity [%]	*N*	Correctly predicted	Sensitivity [%]
US	259	183	70.66	35	21	60.00
scintigraphy	253	146	57.71	33	20	60.61
SPECT-CT	100	56	56.00	10	6	60.00
MRI	104	56	53.85	15	7	46.67
CT	30	9	30.00	11	6	54.55
SVS	5	4	80.00	7	2	28.57
18-F PET-CT	3	3	100.00	1	1	100.00

As shown above in SVS the possibility of correct detection of the parathyroid adenoma depended heavily on previous operations on the thyroid and parathyroid glands (Table 4). It was shown that correct lateral localization was possible in 100% of patients who had not undergone surgery. In 80% of cases, the localization could also be predicted correctly in both horizontal and vertical plane. In the pre-operated patient population, however, the side could only be predicted correctly in 57.1% of cases. The ability to correctly indicate both side and height localization also showed a dramatic decrease from 80.0% to 28.7% in pre-operated patients (Tables 3 and 4).

**Table 4:** Dependence of selective venous blood determination by SVS on previous operations on the thyroid and parathyroid glands. Separate consideration with regard to the preoperative prediction of the correct lateral as well as the correct lateral and vertical localization. Here we see a clear decrease in the sensitivity of the SVS in patients who have already undergone neck surgery

No previous surgery				Previous surgery		
Localization specification	N	Correctly predicted	Percentage of correctly predicted	N	Correctly predicted	Percentage of correctly predicted
Lateral	5	5	100.0%	7	4	57.1%
Lateral and vertical		4	80.0%		2	28.6%

The mean values of the volumes of the parathyroid adenomas showed that larger adenomas are more likely to be correctly detected (Table 5), whereby the parathyroid adenomas correctly detected by MRI (0.97 $cm^3$ on average) were noticeably smaller than in the other procedures. The mean values of the weight showed across all imaging procedures that heavy adenomas were more likely to be localized correctly than lighter ones.

**Table 5:** The dependence of the correct preoperative localization diagnosis of the various imaging procedures on the volume $\left[cm^3\right]$ and weight [g] of the parathyroid adenomas

procedure	Mean value of the volume $\left[cm^3\right]$with incorrect localization	Mean value of the volume $\left[cm^3\right]$with correct localization	Mean value of the weight [g] with incorrect localization	Mean value of the weight [g] with correct localization
US	0.93	1.25	1.16	1.35
CT	1.92	1.37	0.98	1.78
SPECT-CT	0.94	1.32	1.11	1.46
scintigraphy	1.06	1.22	1.2	1.36
SVS	1.8	1.25	0.85	1.28
MRI	0.59	0.97	0.81	1.37
18-F PET-CT	-	1.16	-	0.73

**Fig. 3:**
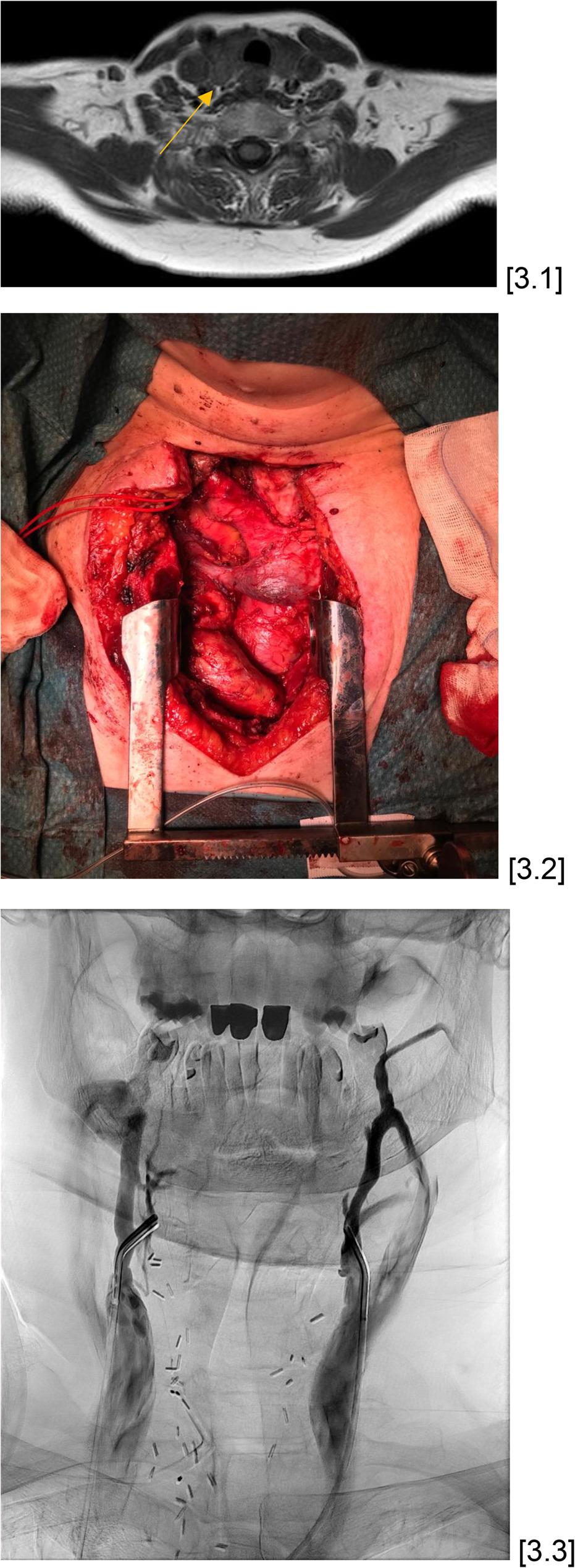
67-year-old female patient suffering from asymptomatic primary hyperparathyroidism with hemithyroidectomy having been performed on the left. Scintigraphic evidence of a parathyroid adenoma on the right side was obtained. However, this suspicion was not confirmed by US, so that a MRI was added, which also confirmed the localization of the adenoma on the right side [3.1]. On the basis of two examinations that agreed on the localization of the adenoma, surgery was subsequently performed, during which the adenoma was confirmed histopathologically in the suspected localization [3.2]. However, the parathyroid hormone did not drop adequately intraoperatively, so that the left side was also explored but without adenoma detection. Postoperatively, another scintigraphy was performed, but this did not show increased tracer uptake. MRI and CT examinations now suggested that the adenoma was located at the level of the junction of the right brachiocephalic vein and the vena cava. A supplementary angiographic examination revealed the highest parathyroid hormone concentration in the superior vena cava [3.3]. In the subsequent re-operation, the adenoma localization consistently suspected on CT and MRI turned out to be merely a lymph node. This was followed by a thymectomy, which histopathologically confirmed an intrathymal located parathyroid adenoma. An adequate decrease in parathyroid hormone from 152.7 pg/ml to 27.4 pg/ml was now observed, which correlates with an intraoperative drop of about 82%. Postoperatively, the parathyroid hormone level remained adequate at 9.5 pg/ml and the calcium level was 2,0 mmol/l. Histopathology confirmed hyperplastic parathyroid tissue compatible with a parathyroid adenoma

Only in 51% of cases the current gold standard consisting of US and scintigraphy was sufficient to make a clear preoperative statement regarding localization; in the other cases, at least one further imaging modality such as MRI was necessary (Fig. 3). A median of three examinations were performed per patient (IQR 2.00–3.00), so that a median of 2 examinations (IQR 1.00–3.00) indicated the correct localization.

The patients were divided into two groups, 36.7% (*N* = 108) were assigned to the SPECT-CT group. The scintigraphy group accounted for 60.5% (*N* = 178) and only 2.7% (*N* = 8) of the patients did not receive a scintigraphic examination. When analyzing the two groups against each other, neither the scintigraphy nor the SPECT-CT group showed a clear superiority in terms of correctly predicted localization (Cohen’s Kappa − 0.019, *p* = 0.721). There was also no advantage in terms of correct localization in the SPECT-CT group compared to the scintigraphically examined patients for concomitant thyroid disease (Cohen’s Kappa − 0.022, *p* = 0.798). With regard to patients who had already undergone neck surgery, no superiority of the SPECT-CT group over the scintigraphy group could be determined in terms of correct localization information (Cohen’s Kappa − 0.007, *p* = 0.963). In our study, no advantage of SPECT-CT could be shown with regard to the correct localization of ectopic parathyroid adenomas compared to conventional scintigraphy. Both groups showed a proportion of 66.67% correctly localized ectopic adenomas.

When analyzing the combinations of different preoperative imaging procedures, the combination of US and scintigraphy accounted for the largest proportion with 34.0% (*N* = 100), followed by the combination of US, scintigraphy and MRI with 21.4% (*N* = 63). A complementary SPECT-CT to US and scintigraphy was performed in 17.0% of cases (*N* = 50). The combination described above was supplemented by an MRI in 10.5% of cases (*N* = 31). The remaining 16 combinations of different imaging techniques used each accounted for less than 5% of the total number of examinations performed (Table 6) and 17.0% (*N* = 50) of the total number of preoperative examinations performed.

**Table 6:** Combination of the preoperative localization diagnostics used. Combinations of imaging procedures with a share of ≥ 5% were examined in more detail with regard to their endpoints

Combination of the imaging performed	Number (*N*)	Percentage (%)
US, scintigraphy	100	34.0
US, scintigraphy, SPECT-CT	50	17.0
US, scintigraphy, SPECT-CT, MRI	31	10.5
US, scintigraphy, MRI	63	21.4
others (total)	50	17.0
US	5	1.7
US, scintigraphy, SPECT-CT, CT	14	4.8
US, scintigraphy, CT	6	2.0
US, scintigraphy, SVS, MRI	1	0.3
US, scintigraphy, SVS, CT	1	0.3
US, scintigraphy, SVS, MRI, CT	4	1.4
US, CT	1	0.3
US, MRI	2	0.7
US, scintigraphy, MRI, CT	1	0.3
US, scintigraphy, 18-F PET-CT	1	0.3
US, scintigraphy, SPECT-CT, CT, MRI	7	2.4
US, scintigraphy, SPECT-CT, CT, MRI, SVS	4	1.4
US, scintigraphy, SPECT-CT, CT, MRI, 18-F PET-CT	1	0.3
US, scintigraphy, SPECT-CT, CT, MRI, 18-F PET-CT, SVS	1	0.3
US, scintigraphy, CT, MRI, 18-F PET-CT	1	0.3

In the following, the combinations of imaging procedures with a share of ≥ 5% of all preoperatively performed combinations of examinations were compared. These included the combination of US and scintigraphy, possibly supplemented by a SPECT-CT or MRI, as well as US and scintigraphy together with a SPECT-CT and MRI.

The investigated combinations of imaging procedures did not provide significantly different results with regard to the adequate postoperative drop in PTH (*p* = 0.426). There were also no differences between the combinations of imaging procedures with regard to the other primary endpoint, namely the adequate drop in calcium levels postoperatively (*p* = 0.256). Furthermore, the main procedures performed also showed no differences in terms of operating time (*p* = 0.660).

A comparison of the histopathologically determined volume of the surgically removed parathyroid adenoma showed no statistically significant difference between preoperatively performed US and scintigraphy compared to the same combination with supplementary SPECT-CT (*p* = 0.657). MRI performed in addition to US and scintigraphy also showed no statistically significant correlation in terms of volume (*p* = 0.051). However, it could be seen that parathyroid adenomas examined by the combination of US, scintigraphy, SPECT-CT and MRI had a statistically significant smaller volume compared to those examined by US and scintigraphy (*p* = 0.004). There was also a statistically significant difference in the volume determined for the combination of US, scintigraphy and SPECT-CT compared to the same combination with an additional MRI (*p* = 0.012). The parathyroid adenomas examined by US, scintigraphy and MRI were also statistically significantly smaller than those examined by US, scintigraphy and SPECT-CT alone (*p* = 0.031). No statistically significant difference was found for the combination of US, scintigraphy and MRI in comparison to the combination of US, scintigraphy, MRI with additionally performed SPECT-CT (*p* = 0.313). In summary, it can be said that, in terms of volume, preoperative localization diagnostics often had to be supplemented by an MRI examination for smaller parathyroid adenomas (Table 7).

**Table 7:** Combination of the preoperatively performed localization diagnostics in comparison to the histopathologically determined volume $\left[cm^3\right]$ and weight [g]

Combination of the localization diagnostics	Mean value of the volume $\left[cm^3\right]$	*N* volume	Std.-deviation volume	Mean value of the weight [g]	*N* weight	Std.-deviation weight
US, scintigraphy	1.25	58	1.36	1.40	69	1.44
US, scintigraphy, MRI	0.82	29	1.28	1.16	36	1.59
US, scintigraphy, SPECT-CT	1.27	31	1.29	1.43	39	1.09
US, scintigraphy, SPECT-CT, MRI	0.52	16	0.64	1.01	19	1.16
others	1.51	33	1.75	1.24	36	0.29

The combination of the most frequently performed preoperative localization diagnostics also revealed statistically significant differences in the histopathologically determined weight (*p* = 0.046). With regard to the weight of the parathyroid adenomas, there was no difference between the combination of US and scintigraphy and the same combination with additional SPECT-CT (*p* = 0.431). However, the combination of US and scintigraphy showed a statistically significant difference compared to a preoperative combination of US, scintigraphy, SPECT-CT and MRI (*p* = 0.045). Even though the mean values of the weights of the parathyroid adenomas were lower with US, scintigraphy and MRI examinations than with US and scintigraphy alone (Table 7), no statistically significant difference could be shown here (*p* = 0.104). In a complementary MRI examination to US, scintigraphy and SPECT-CT, the parathyroid adenomas were also statistically significantly lighter (*p* = 0.036). There were also statistically significant differences regarding the weight between the parathyroid adenomas examined by US, scintigraphy and MRI and those examined by US, scintigraphy and SPECT-CT (*p* = 0.040). In contrast, there was no difference in weights of the patients’ adenomas examined using a combination of US, scintigraphy, SPECT-CT and MRI and those who were examined preoperatively using US, scintigraphy and MRI (*p* = 0.467).

Thus, with regard to the histopathologically determined volume and weight of the parathyroid adenomas, it could also be shown that the standard diagnostics of US and scintigraphy with or without SPECT-CT often did not provide clear results with regard to localization in smaller and lighter adenomas and had to be supplemented by a further examination method in our investigated case, MRI.

In the following the imaging findings have been correlated with biochemical parameters. No statistically significant difference between the most frequently used imaging techniques and patient age (*p* = 0.095) could be found. An analysis of the selected combinations of preoperative localization diagnostics with regard to the preoperative parathyroid hormone level did not reveal any significant differences (*p* = 0.864). In contrast, statistically significant differences were found for the preoperative calcium value between the four groups with different combinations of imaging techniques (*p* = 0.025, η²ₕ = 0.027). Preoperative calcium levels were higher on average when an MRI had to be performed in addition to US and scintigraphy (*p* = 0.009). In contrast, the lowest calcium levels were found in patients who underwent ultrasound, scintigraphy, SPECT-CT and MRI.

If thyroid disease was operated on at the same time, there were no statistically significant differences between the combinations of preoperative imaging performed (*p* = 0.179), even if the ability to correctly localize the parathyroid adenomas decreases for the different procedures (Table 2).

For patients with previous neck surgery on the thyroid or parathyroid gland, there was no superiority between the combinations of preoperative localization diagnostics with regard to operating time (*p* = 0.31). An analysis of the other selected endpoints also showed no significant advantage in pre-operated patients with regard to the adequate postoperative PTH drop (*p* = 0.41) and the adequate postoperative calcium drop (*p* = 0.94).

The preoperative parathyroid hormone level correlated moderately positively with the histopathologically determined adenoma weight (*r* = 0.44, *p* < 0.001). The preoperative parathyroid hormone level correlated somewhat weaker, but still statistically significantly and moderately positively, with the volume of the adenoma (*r* = 0.38, *p* < 0.001). In contrast, preoperatively determined calcium correlated only weakly positively with adenoma weight (*r* = 0.2, *p* = 0.005) and only very weakly positively with adenoma volume (*r* = 0.17, *p* = 0.024). According to Spearman’s rho, a statistically significant moderate positive correlation was also found between preoperative parathyroid hormone levels and preoperative calcium levels (*r* = 0.45, *p* < 0.001).

**Fig. 4:**
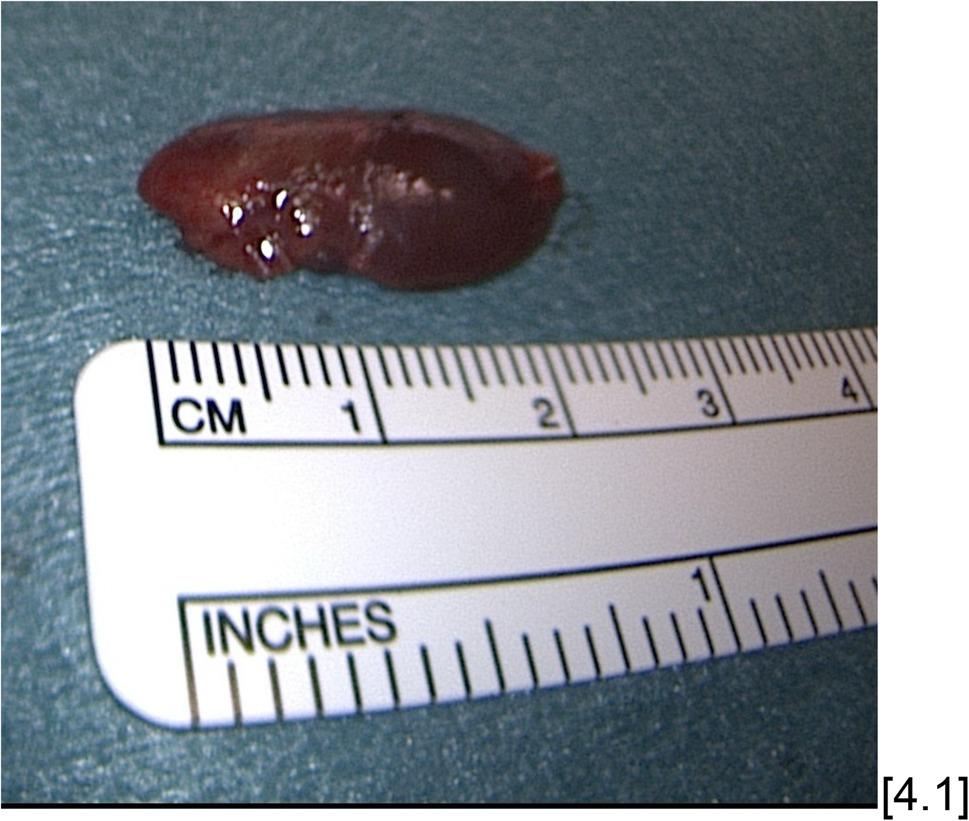
38-year-old female patient with primary hyperparathyroidism discovered by chance during a thyroid examination and after urolithiasis. US and scintigraphy initially revealed an adenoma localization on the central right side. However, the operation then had to be aborted without success and a selective venous blood sample was taken the following day, which revealed a caudal left adenoma localization. A parathyroid adenoma measuring approximately 2.2 x 1.0 x 0.4 cm and weighing 0.6 g was removed during the reoperation [4.1]. There was also an adequate drop in parathyroid hormone of about 91% from 243.5 pg/ml to 22.9 pg/ml and histopathological evidence of lipomatous parathyroid tissue, consistent with an adenoma. Postoperatively, the parathyroid hormone level remained adequate at 28.5 pg/ml and the calcium level was 2.3 mmol/l

**Fig. 5:**
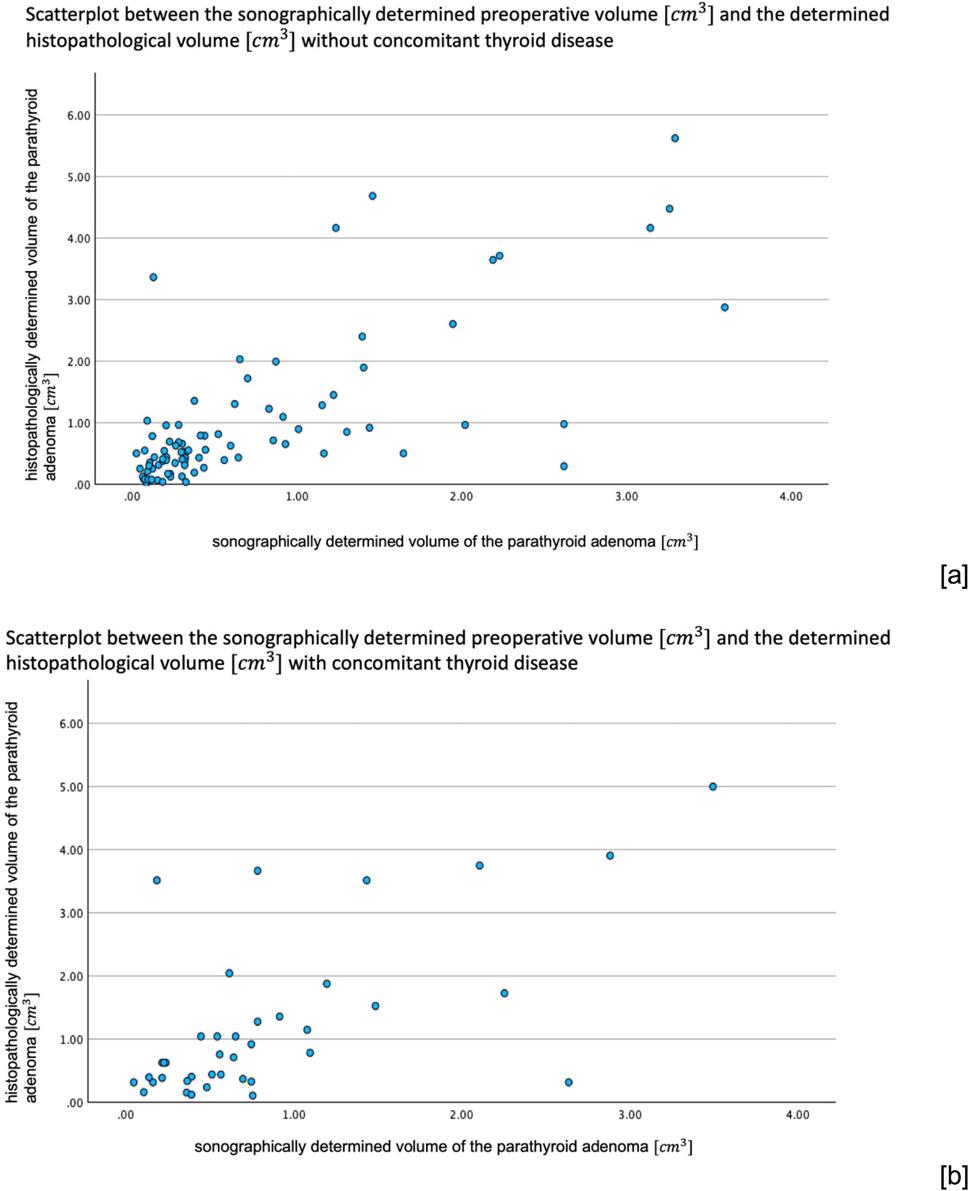
Correlation between the sonographically determined preoperative volume $\left[cm^3\right]$ and the determined histopathological volume $\left[cm^3\right]$. **a** This graph shows the very strong correlation between the sonographically predicted volume of the parathyroid adenoma and the actual histopathologically determined volume in the absence of concomitant thyroid disease worthy of surgery. **b** On the other hand, this graph shows the still strong correlation between the sonographically determined volume and the histopathologically determined volume in the presence of concomitant thyroid disease

In addition, we investigated how well the preoperative sonographic volume of the parathyroid adenoma correlates with the actual histopathologically determined volume (Fig. 4). Without concomitant thyroid disease requiring surgery, there was a very strong correlation between the sonographically determined volume and the histopathologically determined volume with a Spearman correlation coefficient of 0.702 (*p* < 0.001) (Fig. 5a). With concomitant thyroid disease, the correlation deteriorated to a still strong correlation with a Spearman correlation coefficient of 0.600 (*p* < 0.001) (Fig. 5b). Due to insufficient case numbers of CT and MRI findings with preoperatively determined volumes of parathyroid adenomas, these imaging modalities could only be examined as a whole with regard to their correlation to the histopathologically determined volumes, but not separately according to the presence of thyroid disease. For MRI, there was a strong correlation as well (*p* = 0.009) with a Spearman correlation coefficient of 0.666. For CT, a very strong correlation (*p* = 0.042) with a Spearman correlation coefficient of 0.829 could be found. 

## Discussion

In contrast to many previous studies that were performed under standardized research conditions, our analysis reflects data collected in routine clinical settings across multiple hospitals and outpatient practices. This approach allows an evaluation of diagnostic accuracy in the context of routine, real-world clinical practice.

The primary endpoint of postoperative normalization of parathyroid hormone levels after MIVAP was achieved in 94.2% of patients undergoing surgery. Postoperative normalization of calcium levels could be achieved in 78.6% of patients.

The combination of US and scintigraphy as imaging procedures performed as standard were able to provide clear information regarding the localization of the adenoma in 51% of cases. In our study, supplementary preoperative imaging was performed in all cases of discordant findings in sonography and scintigraphy in routine clinical practice. Other approaches assume that minimally invasive parathyroidectomy (MIP) is also possible in these cases of inconsistent findings without extended imaging, using only intraoperative parathyroid hormone monitoring. However, according to Canu et al. this results in significantly longer operating times and a more frequent need for BNE [20].

In our department standard diagnostics were most frequently supplemented by MRI. On average, three examination modalities (triple localization methods) had to be performed per patient (IQR 2.00–3.00), with MRI as the most common supplemented procedure, so that in the end a median of two examinations (IQR 1.00–3.00) consistently indicated the correct localization.

In our study, US showed the best results in clinical routine with a 78.82% rate of correctly predicted preoperative localization of parathyroid adenomas without concomitant thyroid disease in accordance with the prescribed pooled sensitivity of 76,1% [21]. With concomitant thyroid disease requiring surgery, this value decreased noticeably to 56.45%, but still showed the best results of the examinations carried out in routine clinical practice. In addition, the correlation between the sonographically determined volume of the adenoma and the histopathologically determined volume also decreased in the presence of concomitant thyroid disease. Such a deterioration of preoperative imaging with concomitant thyroid disease has been described for US and scintigraphy [22]. This is an important limitation, as 42.2% of all patients in our study underwent thyroid and parathyroid surgery at the same time. Beyond that, US imaging has its limits when it comes to detecting smaller parathyroid adenomas and ectopic adenomas below neck level VI or multi-gland disease, as well as with intrathyroidal localization [23]. Another limitation of US is its dependence on the examiner, which is why a combination of US and scintigraphy is the current gold standard and why it should be performed by specialist head and neck radiologists with extensive experience in parathyroid imaging [24]. However, US has proven to be useful for the detection and localization of parathyroid adenoma in an intraoperative setting [25].

Planar scintigraphy has subsequently been expanded to three-dimensional SPECT imaging, which improves the detection of posterior or retro-oesophageal adenomas. The combination of SPECT with CT further enhances anatomical correlation and facilitates differentiation from surrounding tracer-avid structures [24]. Our study showed that the sensitivity of scintigraphy in clinical practice, at 58.0%, is lower than the reported pooled sensitivity of 78.9% (64–90.6%). This reduction in sensitivity in broad clinical practice could be due to the fact that our data originate from different centres with different protocols and equipment [21]. However, within our cohort neither the scintigraphy nor the SPECT-CT group showed a clear superiority in terms of correctly predicted localization. The sensitivity of SPECT-CT in our study was 56.4%, considerably lower than the 75.1% reported in previous publications [26].

99 m-Tc-sestamibi is based on the visualization of mitochondria-rich cells and thus hyperfunctional glandular tissue [27]. There are inconsistent studies regarding the dependence of scintigraphy on the volume and weight of the parathyroid adenoma to be visualized. Earlier studies have shown that scintigraphy tends to yield positive results with an increasing weight [28, 29]. Other studies, however, were unable to show any correlation between tracer uptake and the volume of the adenoma [30]. However, our findings indicate heavier and larger parathyroid adenomas were more likely to be correctly detected both in scintigraphy and in SPECT-CT.

MRI protocols typically include axial T1- and T2-weighted sequences before and after contrast application, often combined with fat-suppressed imaging to improve visualization of parathyroid lesions [31]. The sensitivities of MRI specified in the EANM guidelines vary greatly between different studies, ranging from 43.4% [32] on a 1.5 T device to 97.8% on a 3 T device [33]. In our study, the sensitivity of MRI was only 52.9%, which is in the lower range indicated. This result could be primarily due to the fact that, unlike Argiró et al., our study was conducted as a multicentre study involving different clinics and practices, meaning that different MRI protocols, magnetic field strengths and devices were used [33]. On the one hand, this represents a limitation of our study that no further differentiation was made between the different protocols, field strengths and devices used. On the other hand, however, the aim of our study was also to highlight the difference between the values determined under study conditions and the results from clinical practice or routine use. In contrast to the standardized procedures used in study conditions, the range of protocols used in routine clinical practice, particularly in MRI examinations, is significantly greater. In addition, the MRI examination in broad clinical application is only a second-line examination, which means that it was only used in more complex cases when US and scintigraphy could not provide a clear statement regarding the localization, which could also explain the lower sensitivity compared to the literature.

Overall, uncertainties remain, as the appearance of parathyroid adenomas on MRI is similar to that of lymph nodes or ectopic thyroid tissue. Analogous to 4D-CT, there are also 4D-MRI protocols which also make use of the hypervascular behavior of parathyroid adenomas. Dynamic MRI has a sensitivity of 91% in a pre-operated patient population. Another optimization is magnetic resonance angiography, which has a sensitivity of 93% [31]. As reported in literature, patients with concomitant thyroid disease showed a reduction in the sensitivity of the MRI, although this was not significant [34]. To obtain a good image of parathyroid adenomas, fat suppression is essential, but this is difficult to achieve in MRI of the neck region and the mediastinum, as the proximity to the lungs and upper airways leads to an inhomogeneity of the magnetic field. The DWI sequence appears to improve the ability to correctly detect parathyroid adenoma on MRI. By measuring the ADC values, parathyroid adenomas may be differentiated from lymph nodes and the thyroid gland due to higher diffusion properties [35, 36]. In addition the sensitivity can be further increased by using a 3T MRI, as this has increased spatial resolution and signal-to-noise-ratio, which potentially allows the detection of smaller parathyroid adenomas, however, can lead to more distortion [33, 35]. Good visualization of parathyroid adenomas on MRI thus remains challenging as our evaluation shows as there is no standardized MRI protocol up to date. Further research with the establishment of standardized examination protocols will be necessary here in order to improve the overall sensitivity of MRI examinations regardless of where they are performed.

In our study, the sensitivity of correctly predicted localizations on CT was only 47.1% compared to the literature, where sensitivity for 4D-CT is reported to be 76.5% [37] up to 92.5% for single-gland disease [26]. In our study, the sensitivity of CT was noticeably lower than the values reported in the literature. This difference may be explained by the heterogeneous imaging protocols, scanner types, and the broader clinical setting of our study, as CT was mainly used as a second-line modality after inconclusive first-line imaging.

4D-CT provides both anatomical and dynamic information by evaluating contrast enhancement across several imaging phases. Parathyroid adenomas typically appear hypodense compared with thyroid tissue on non-contrast images and show rapid arterial enhancement followed by washout in delayed phases, which helps to distinguish them from lymph nodes [38].

Despite the higher radiation exposure, 4D-CT can be particularly useful in complex cases, including reoperative patients or when other imaging modalities fail to localize the pathological gland. 4D-CT may be performed with PET-CT tracers to achieve even greater diagnostic accuracy [10, 25]. Further studies with larger patient numbers are required to better define its role in routine clinical practice.

Overall, SVS showed a sensitivity of 50% in our study, notably lower than the 74% reported in the literature [39]. Among patients without prior neck surgery, sensitivity increased to 80%, which corresponds to the value stated in the literature. This could be explained by anatomically altered vascular courses after previous operations, a fact which is also consistent with other studies [40]. Given the small number of cases (*n* = 12), this finding further illustrates the minor role of this invasive method in everyday clinical practice. SVS is an invasive diagnostic tool that has numerous potential risks such as bleeding, aneurysms, fistulas or infections. However, the complications of SVS are rare and none of the patients in our study suffered such a complication from the examination. Since SVS is performed as an invasive examination in patients with negative, equivocal or inconsistent findings in the non-invasive examinations, the alternative to this invasive examination would be performing a BNE, which, however, is also associated with numerous potential complications. The dose of radiation in the SVS is approximately half that of a 4D-CT [41]. Our findings suggest that the indication for this invasive examination should be rather restrictive especially in patients who have already undergone surgery.

In our cohort 18-F PET-CT demonstrated a sensitivity of 100%, successfully identifying smaller and lighter adenomas, including patients with concomitant thyroid disorders. Nevertheless, its use in routine clinical practice in our study is limited to four cases due to high costs. Reported sensitivity in the literature is slightly lower, at 96.2% [42]. The 18-F PET-CT seems to be better able to detect smaller parathyroid adenomas than SPECT-CT. This can be explained by the fact that, in contrast to SPECT-CT, 18-F PET-CT has a better spatial resolution [43]. Alharbi et al. suggested that 18-F uptake in parathyroid adenomas is strongly correlated with serum parathyroid hormone levels [44]. In this context a further correlation between imaging findings in 18-F PET-CT and biochemical parameters would be useful in a larger cohort.

However, the examination procedures must always be considered in their combination, since, for example, matching localizations in US and scintigraphy are associated with a high positive predictive value [45]. This prompted us to analyze the most common combinations of preoperative imaging modalities with a total share of greater than or equal to 5% in more detail. These included the combination of US and scintigraphy, possibly supplemented by a SPECT-CT or MRI, as well as US and scintigraphy together with a SPECT-CT and MRI. In terms of volume and weight, preoperative localization diagnostics often had to be supplemented by an MRI examination for smaller and lighter parathyroid adenomas.

No statistically significant differences were found between the four most commonly used preoperative imaging techniques in terms of preoperative parathyroid hormone levels. In contrast, the lowest calcium levels were found in the most complex of the four most commonly performed preoperative imaging procedures, consisting of US, scintigraphy, SPECT-CT and MRI. This suggests that adenomas with lower secretory activity and consequently lower calcium levels tend to appear smaller or less characteristic in imaging and therefore more often require additional imaging procedures. In contrast, the group undergoing US, scintigraphy and MRI showed the highest calcium levels. This could be explained by the fact that in this constellation, MRI was often used specifically to confirm findings that were already highly suspicious based on biochemical and scintigraphic evidence, rather than to supplement a difficult diagnosis. Overall, it can therefore be concluded that higher preoperative calcium levels do not necessarily correlate with easier imaging detection, but lower calcium levels are more often associated with greater diagnostic effort. The analysis of these parameters would also be useful in future for CT, 18-F PET-CT, and SVS with a larger number of cases for the corresponding modalities.

There is a significant but varying correlation between the mass of parathyroid adenomas and biochemical parameters. While preoperative parathyroid hormone correlated moderately with adenoma weight (*r* = 0.44; *p* < 0.001) and adenoma volume (*r* = 0.17, *p* = 0.024), calcium showed only weak, albeit significant, correlations with adenoma weight (*r* = 0.20; *p* = 0.005) and volume (*r* = 0.17; *p* = 0.024). This suggests that systemic calcium concentration is regulated more strongly by homeostatic mechanisms and is less directly dependent on tumor mass than parathyroid hormone concentration. However, previous studies have shown that parathyroid hormone levels also correlate with patient weight and that thiazide diuretics and lithium may increase calcium levels [46]. The expected positive correlation between preoperative parathyroid hormone and calcium (*r* = 0.45; *p* < 0.001) confirms the functional consistency of the data collected.

**Fig. 6:**
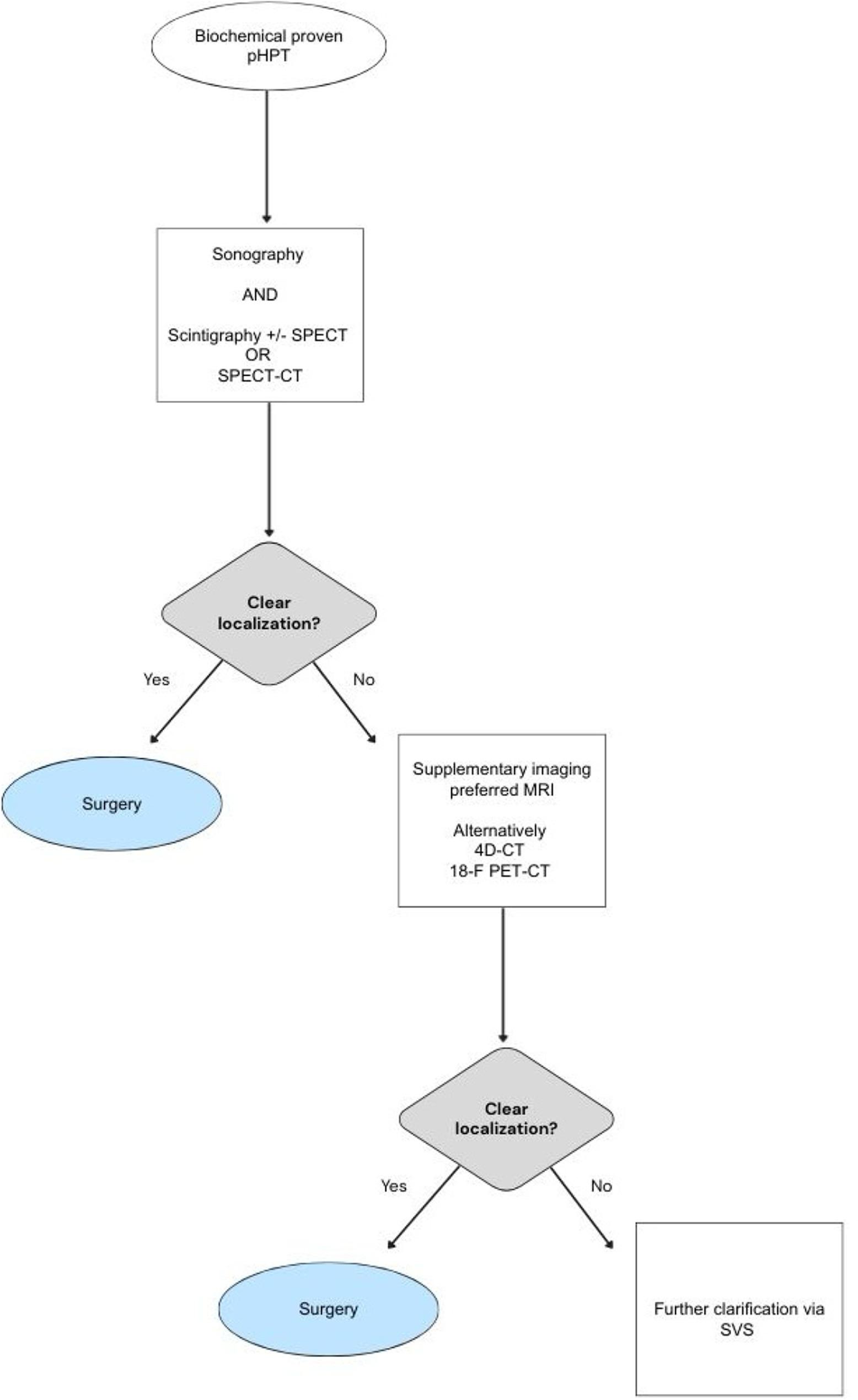
Diagnostic imaging algorithm in pHPT. Flowchart illustrating the stepwise diagnostic imaging approach in patient with biochemically proven pHPT. First-line imaging includes cervical US and scintigraphy with or without SPECT or SPECT/ CT. In equivocal or discordant cases supplementary imaging modalities including preferred MRI, alternatively 4D-CT or 18-F PET-CT are employed to further guide surgical planning. If localization remains inconclusive, additional clarification via SVS may be considered

In conclusion in broad clinical application for preoperative imaging, ultrasound and scintigraphy should be performed first. In case of inconclusive results, imaging should be supplemented with cross-sectional imaging, preferably MRI (Fig. 6). The indication for an invasive examination such as SVS should be made very restrictively, especially in patients who have already undergone surgery. The 18-F PET-CT delivered very promising results, but plays a minor role in routine clinical practice for cost reasons. Further studies will be necessary to confirm the potential of this examination modality in broad clinical application with higher case numbers. Furthermore, future studies should pay particular attention to the best preoperative localization diagnostics for multiglandular disease.

### Limitations of study

The study was conducted as a retrospective study, resulting in quite large differences with regard to the group size of the examinations performed. The examinations were carried out with different devices, protocols and examiners, which could have an influence on the possibility of correctly predicting the preoperative localization as the sensitivity of the same imaging modality varies greatly between different institutions according to the frequency with which it is performed [25].

A limitation of this study is the absence of a standardized imaging protocol, particularly for MRI, CT, SPECT-CT, and scintigraphic examinations. However implementing such uniformity would not accurately reflect real-world clinical practice.

Consistently, the scintigraphies were performed using either Tc-99 m sestamibi or tetrofosmin and were not further quantified according to the tracer used. In addition, the MRI examinations performed were not analyzed in detail with regard to the field strength of the device used and the sequences performed. This also applies to the different protocols between the various institutions for 4D-CT, SPECT, as well as SPECT-CT and MRI. Depending on the different protocols used at the SVS, the possibility of preoperative localization may also vary here across different institutions.

The lack of statistical significance of the different preoperative imaging modalities on the intra- to preoperative drop in parathyroid hormone could be explained by the surgical procedure. If the parathyroid hormone drop is insufficient, further exploration is performed until an adequate parathyroid hormone drop is achieved. Only in a few cases the operation was cancelled despite an inadequate drop in parathyroid hormone. This results in an adequate drop in parathyroid hormone for both the group with and without correct imaging and therefore limited assessability with regard to this endpoint.

A further limitation of the study results from the exclusion of patients with multiglandular disease This selection aimed to ensure consistent preoperative imaging-pathology correlation but may limit generalizability. Multiglandular parathyroid disease accounts for 15–20% of patients with pHPT and places special demands on imaging [10]. Further studies will be necessary in this regard to address the difficulties of localizing multiple hyperplastic lesions.

Due to small number of cases, 4D-CT, 18-F PET-CT and SVS could not be analyzed statistically in greater detail.

## Conclusion

This study demonstrates that the combination of US and scintigraphy as primary imaging modalities is a very effective strategy to localize parathyroid adenomas for the majority of patients and is associated with a high minimally invasive parathyroidectomy success rate in a routine setting in patients with surgical criteria of a thyroid center.

The current gold standard of US and scintigraphy was able to provide a clear statement with regard to a clear preoperative prediction of the localization in 51% of cases and had to be supplemented by further imaging modalities in the remaining cases. Nevertheless, there remains a significant cohort of patients in whom additional techniques have to be required for further preoperative localization, with MRI being used most frequently.

In contrast to the sensitivities reported in the literature mostly under controlled study conditions the sensitivities were noticeably lower for scintigraphy, SPECT-CT, MRI and CT, which could be due primarily to the widely varying examination protocols and equipment used in broad clinical application. The sensitivities of US, scintigraphy, SPECT-CT and MRI were all impaired in the presence of concomitant thyroid disease. The larger the volume and the higher the weight of the resected adenoma, the easier it was to detect parathyroid adenomas preoperatively. In our study, 18-F PET-CT, showed the highest sensitivity independent of previous operations and concomitant thyroid diseases. However, it is rarely used in clinical applications due to a lack of availability and cost reasons. The sensitivity of the SVS was clearly dependent on previous thyroid and parathyroid operations.

In clinical practice, ultrasound and scintigraphy should be performed first. If the results are inconclusive, supplementary cross-sectional imaging should be performed, preferably MRI. The indication for SVS should be made very restrictive, especially in patients who have already undergone surgery. Following this algorithm, the primary endpoint of postoperative normalization of PTH levels in our study could be achieved in 94.2% of patients. The 18-F PET-CT delivers very promising results but has not yet found its way into widespread clinical use. Further studies with higher case numbers are needed in the future to confirm the promising potential of this imaging technique in broad clinical application. In addition, further investigations are also needed for multi-glandular diseases to confirm a corresponding diagnostic algorithm. 

## Data Availability

Most of the data are included in this published article. Supplementary data are not openly available due to reasons of sensitivity and are available from the corresponding author upon reasonable request. Data are located in controlled access data storage at Johanniter Hospital Bonn.

## Abbreviations

ADC: Apparent diffusion coefficient
BNE: Bilateral neck exploration
CT: Computed tomography
DWI: Diffusion-weighted magnetic resonance imaging
EP: Endoscopic parathyroidectomy
18-F PET-CT: 18-fluorocholine positron emission tomography combined with computed tomography
MIP: Minimally invasive parathyroidectomy
MI-RP: Minimally invasive radio-guided parathyroidectomy
MIVAP: Minimally invasive video-assisted parathyroidectomy
MRI: Magnetic resonance imaging
OMIP: Open minimally invasive parathyroidectomy
PET: Positron emission tomography
pHPT: Primary hyperparathyroidism
PTH: Parathyroid hormone
SPECT: Single-photon emission computed tomography
SPECT-CT: Single-photon emission computed tomography combined with computed tomography
SVS: Selective venous blood sampling
99m Tc sestamibi: Technetium-99 m bound to six methoxyisobutylisonitrile ligands
TSE sequence: Turbo-spin-echo sequence
US: Cervical ultrasound
VATS: Video-assisted thoracoscopic surgical (VATS)
